# Collagen/PCL Nanofibers Electrospun in Green Solvent by DOE Assisted Process. An Insight into Collagen Contribution

**DOI:** 10.3390/ma13214698

**Published:** 2020-10-22

**Authors:** Dalila Miele, Laura Catenacci, Silvia Rossi, Giuseppina Sandri, Milena Sorrenti, Alberta Terzi, Cinzia Giannini, Federica Riva, Franca Ferrari, Carla Caramella, Maria Cristina Bonferoni

**Affiliations:** 1 Department of Drug Sciences, University of Pavia, Viale Taramelli 12, 27100 Pavia, Italy; dalila.miele@unipv.it (D.M.); laura.catenacci@unipv.it (L.C.); silvia.rossi@unipv.it (S.R.); giuseppina.sandri@unipv.it (G.S.); milena.sorrenti@unipv.it (M.S.); franca.ferrari@unipv.it (F.F.); carla.caramella@unipv.it (C.C.); 2 Institute of Crystallography, CNR-IC, Via Amendola 122/O, 70126 Bari, Italy; alberta.terzi@ic.cnr.it (A.T.); cinzia.giannini@ic.cnr.it (C.G.); 3 Department of Public Health, Experimental and Forensic Medicine, University of Pavia, via Forlanini 2, 27100 Pavia, Italy; federica.riva01@unipv.it

**Keywords:** electrospun nanofibers, collagen, polycaprolactone, design of experiments

## Abstract

Collagen, thanks to its biocompatibility, biodegradability and weak antigenicity, is widely used in dressings and scaffolds, also as electrospun fibers. Its mechanical stability can be improved by adding polycaprolactone (PCL), a synthetic and biodegradable aliphatic polyester. While previously collagen/PCL combinations were electrospun in solvents such as hexafluoroisopropanol (HFIP) or trifluoroethanol (TFE), more recently literature describes collagen/PCL nanofibers obtained in acidic aqueous solutions. A good morphology of the fibers represents in this case still a challenge, especially for high collagen/PCL ratios. In this work, thanks to preliminary rheological and physicochemical characterization of the solutions and to a Design of Experiments (DOE) approach on process parameters, regular and dimensionally uniform fibers were obtained with collagen/PCL ratios up to 1:2 and 1:1 w/w. Collagen ratio appeared relevant for mechanical strength of dry and hydrated fibers. WAXS and FTIR analysis showed that collagen denaturation is related both to the medium and to the electrospinning process. After one week in aqueous environment, collagen release was complete and a concentration dependent stimulatory effect on fibroblast growth was observed, suggesting the fiber suitability for wound healing. The positive effect of collagen on mechanical properties and on fibroblast biocompatibility was confirmed by a direct comparison of nanofiber performance after collagen substitution with gelatin.

## 1. Introduction

Reparative medicine is an emerging field thanks to the recent increasing availability of artificial substrates or scaffolds capable to support cell growth and therefore to restore or recover damaged tissues, among which skin wounds or burns. Besides biocompatibility, key feature of suitable substrates is their ability to mimic the extracellular matrix (ECM) as structural framework and regulation of cell-response behavior [1,2,3]. Natural polymers commonly found in extracellular matrix as proteins (collagen, elastin, fibronectin) or glycosaminoglycans (chondroitin sulfate, hyaluronic acid) are mostly employed to best mimic the physiological environment [1,4,5,6]. In particular, collagen is the main fibrous protein of human body and, within ECM, provides cell migration, adhesion, and differentiation, promoting angiogenesis [7,8,9]. Due to its excellent biocompatibility and biodegradability and to its weak antigenicity, collagen represents one of the most used natural biomaterials in skin replacement, bone substitutes, and artificial blood vessels and valves [10,11]. It can be processed by cross-linking, freeze drying, or electrospinning to obtain hydrogels, membranes, matrices, and fibers [11]. Electrospinning of collagen has been widely studied in literature [12,13]. However, in most cases the use of organic solvents causes collagen denaturation resulting in gelatin nanofibers [14].

Gelatin derives from collagen thermal denaturation and hydrolysis, mediated by acid or base condition. Its extensive use as an alternative of collagen in biomedical field is related to its low-cost and easy achievement. The amino acid compositions of collagen and gelatin are almost identical; however, other characteristics such as secondary structure, isoelectric point, and molecular weight distribution are different, affecting the physical properties of the 3D-tissue substitutes [15,16].

The mechanical and morphological stability of both collagen and gelatin nanofibers can be improved by combining the protein with different synthetic biodegradable polymers such as polylactic acid (PLA), polylactic-polyglycolic acid (PLGA), or poly-ε-caprolactone (PCL) in the electrospun solution [17]. On the other way, the presence of collagen improves wettability, biocompatibility and cell recognition of the fibers. A relevant role has been claimed to the presence in collagen of chemotactic sequences able to stimulate the formation of granulation tissue essential for wound healing. It is also worth noting that the presence of both triple helical integrity and the RGD (Arg-Gly-Asp) ligand explosion are selectively recognized by specific integrin on the cellular membrane, leading to cell attachment [11,18,19,20,21,22]. Different wound dressings based on collagen have been for this reason proposed to cover burns and wounds, as they help in promoting cell growth and activating inflammatory phase of wound healing [23]. PCL is an FDA-approved synthetic, hydrophobic, and biodegradable aliphatic polyester. However, due to a lack of cell-recognition sites and poor hydrophilic character, PCL has shown non optimal cell adhesion, proliferation, and migration when utilized as cell growth support. This limit can be properly overcome by the combination of PCL with either collagen or gelatin [20,24,25]. Therefore, a synergistic effect is achieved, and this combination not only allows to significantly decrease PCL hydrophobicity, but also has a favorable influence on cellular response of the scaffold [11,17,22]. In previous studies collagen/PCL combination has been electrospun by using organic, toxic, and expensive solvents such as 1,1,1,3,3,3-hexafluoro-2-propanol (HFP) or 2,2,2-trifluoroethanol (TFE) [19,20]. In more recent papers, nanofibers based on collagen/PCL have been electrospun in benign acidic aqueous solutions [21,26,27,28]. The choice of acetic acid as solvent can promote the exploitation of inexpensive renewable feedstocks, because acetic acid can be obtained from cellulosic biomass such as agricultural residues [29]. Moreover, the increasing concern, especially in EU regulations, for the impact of chemicals on health and environment encouraged the pharmaceutical industries to substitute toxic or hazardous solvents with green ones [30]. Furthermore, several works demonstrated the fundamental contribution of the solvent to the collagen structure, showing in particular the strong denaturation effect of fluoroalcohols and TFE solvents on the triple helical structure during solvent casting processes, in contrast with the results reported for acetic acid [12,31,32]. However, it appears from the literature that some work is still necessary to improve the morphology of collagen/PCL nanofibers especially based on relatively high collagen concentration (up to 50% w/w). In the present work to improve nanofiber morphology, PCL, collagen, and collagen/PCL, randomly collected nanofibers in acetic acid 90% v/v aqueous solution, were developed by a two-step approach. During the first phase the peptide and polymer concentration in electrospun solutions were studied by a rheological study aimed to determine the relationship between polymer concentration and entanglement behavior [33,34,35,36]. Therefore, the electrospinning process parameters were studied by means of a DOE approach. This represents a powerful instrument for a systematic understanding of the relevance of critical parameters and their interactions on product characteristics. Morphology and physical properties of PCL, collagen, and collagen/PCL nanofibrous membranes were assessed by SEM analyses, wettability tests and tensile strength. The contribution to collagen degradation of the use of green but anyway strongly acidic environment and of the mechanical effect of electrospinning process was assessed by Wide Angle X-ray Scattering (WAXS) analyses and FTIR spectroscopy. Collagen release during the exposition to aqueous environment was investigated. In the perspective of a potential application of the collagen/PCL fibers in wound healing, the contribution of collagen to mechanical properties of nanofibrous membranes both dry and in wet form was considered. Biocompatibility on normal human dermal fibroblasts and cell proliferation and adhesion were visualized by scanning electron and laser confocal microscopy. Finally, to verify whether the use of collagen, in spite of his degradation during the preparation process, could maintain some advantages with respect to gelatin, similar collagen/PCL and gelatin/PCL nanofibrous membranes were compared in terms of mechanical properties and biocompatibility.

## 2. Materials and Methods 

### 2.1. Materials

Polycaprolactone (PCL-Mw 80,000 Da) and gelatin type B were purchased from Sigma Aldrich (Milan, Italy). Soluble native collagen was purchased from Kelisema S.R.L. (Como, Italy) as 1% (w/v) aqueous solution. Glacial acetic acid from Carlo Erba (Milan, Italy) was employed as solvent.

### 2.2. Methods

#### 2.2.1. Preparation of Protein and Polymer Solutions

Native soluble collagen was dialyzed for 24 h in a dialysis bag (cut-off 12–14 kDa dialysis tubes, Emanuele Mires Ø = 36/32”–28.6 mm) to remove the preservative agents; then the protein solution was freeze-dried for 48 h. The solid residue was dissolved at different concentrations in acetic acid 90% (v/v) aqueous solution obtained by diluting glacial acetic acid with distilled water. Poly-caprolactone (PCL) was solubilized at various concentrations in acetic acid 90% (v/v) aqueous solution, in a water bath at 37 °C and under magnetic stirring (400 rpm) for 4 h; subsequently the polymer solution was sonicated for 30 min at the same temperature. Collagen/PCL and gelatin/PCL mixtures were obtained in 90% (v/v) acetic acid by adding, under magnetic stirring, the protein to the PCL solution in two different weight ratios, 1:1 and 1:2, respectively.

#### 2.2.2. Electrospinning Process

Nanofibers were obtained by using the STKIT-40 (Linari Engineering, Pisa, Italy). A 10 mL glass syringe with a stainless-steel needle (Ø = 0.8 mm, L = 15 mm) was filled with the proper solution and a volumetric pump (Razel R99-E, Saint Albans, VT, USA) was employed for conveying. A high voltage generator (5–40 kV) charged the solution by applying a potential difference between the metal needle and a static collector covered with an aluminum foil. The operating spinning conditions, such as flow rate, voltage applied, and needle-collector distance were modulated according to the characteristics of the polymer solutions to obtain continuous fibers without beads. The process was carried out at atmospheric pressure, maintaining temperature and relative humidity (RH) within an optimal set interval (24–30 °C, 22–30% RH) to allow the solvent evaporation and avoid beads formation.

#### 2.2.3. Rheological Characterization of Polymer and Protein Solutions

Rheological analyses were carried out using a rotational rheometer (Rheostress 600, Haake, Enco, Spinea, Verona, Italy). Solution viscosity (η) was measured at 25 °C and at increasing shear rates in the range 10–1000 s^−1^. Three replicates were performed for each solution.

Solutions at different concentrations of PCL and collagen/PCL in a 1:1 weight ratio were prepared in acetic acid 90% (v/v) aqueous solution with the aim of identifying the critical entanglement concentration (CEC). This represents the lowest concentration at which the solution shows a non-Newtonian behavior as a consequence of the polymeric chain entanglement [33,34]. CEC is assumed as the minimum concentration needed to obtain nanofibers [37].

For each concentration analyzed a shear stress (τ) versus shear rate (s^−1^) plot was processed to extrapolate zero shear viscosity (η_0_) and infinite shear viscosity (η_∞_) values. These values were plotted against % w/w polymer concentration; after log–log transformation of the values on the axes, the CEC was determined as the intersection point of the two straight lines [33].

#### 2.2.4. Conductivity and Surface Tension Measurements

Conductivity analyses were performed using FiveGoTM-Mettler Toledo conductimetry (Mettler Toledo, Milan, Italy). Before the analyses, the instrument was calibrated at room temperature (about 25 °C) with a standard of 1413 μS/cm. Three replicates were performed for each solution. 

Dynamic surface tension measurements were carried out by means of an automatic tensiometer (DyneMaster DY-300; Kyowa Interface Science Co. Ltd., Saitama, Japan) at 25 °C. The analyses were performed by recording a surface tension value every 3 s up to 300 s in a measurement range from 0 to 100 mN/m. Three replicates were considered for each solution.

#### 2.2.5. Experimental Design

A Design of Experiment (DOE) approach was used to assess the influence of the electrospinning parameters on nanofiber dimensions and morphology for both collagen/PCL electrospun mixtures (2:1 and 1:1 weight ratio). A full factorial design 2^3^ was considered, whose factors were the distance between the tip and the collector (cm), the voltage applied (kV) and the pump flow of the syringe (ml/h). Two levels (−1 lower level; +1 higher level) were considered for each factor. The low (−1) and high (+1) levels considered for each of the three factors were: 15 and 25 cm for distance, 20 and 30 kV for voltage, 0.4 and 0.8 mL/h for flow. Eight experiments were therefore carried out to complete the 2^3^ full factorial design (Table 1). Three additional experiments (C.P.) were performed at central points (20 cm, 25 kV, and 0.6 mL/h) of the space design to evaluate the experimental variability.

The experimental design was executed in randomized order. The following response variables were considered: Mean fiber dimensions (nm), number of beads in a defined area and coefficient of variation of the dimensions (% CV). The response variables were acquired from nanofiber SEM images. The statistical analysis of the data was carried out with a statistical software package (Statgraphics Centurion 16, Statistical Graphics Co., Rockvillle, MD, USA).

#### 2.2.6. Characterization of the Nanofibers

##### SEM Analysis

Scanning electron microscopy (SEM) images were acquired at room temperature by using a high-resolution FE-SEM scanning electron microscope (Tescan Mira3 XMU series, Brno-Kohoutovice, Czech Republic); a high voltage (20 kV) and a high vacuum (6 × 10^2^ Pa) were applied. Fiber diameters were analyzed and processed by SEM photomicrographs using ImageJ 2.0 Software (ImageJ, U.S. National Institutes of Health, Bethesda, MD, USA). Measures were performed on at least 60 fibers of each type.

##### FTIR Analysis

FT-IR spectra were recorded using a Perkin Elmer Spectrum One spectrophotometer was used (Perkin Elmer, Monza, Italy) equipped with a MIRacle^TM^ ATR accessory (Pike Technologies, Madison, WI, USA) with a ZnSe crystal cell. The samples have been analyzed at room temperature and the analyzes were recorded in the 4000–650 cm^−1^ range with a resolution of 4 cm^−1^.

##### WAXS Analysis

The wide-angle X-ray scattering experiments were performed at the X-ray Micro Imaging laboratory (XMI-LAB, Institute of Crystallography (IC), National Research Council, Bari, Italy) equipped with a Fr-E + SuperBright rotating copper anode micro-source (λ = 0.154 nm, 2475 W) which is coupled by a multilayer focus optics (Confocal Max-Flux CMF 15-105) to a SAXS/WAXS three pinhole camera. WAXS analysis data were collected using an image detector (IP) (250 × 160 mm^2^, with 100 µm effective pixels) and an offline RAXIA reader. Samples were directly placed on a sample holder, the detector was located 10 cm away from it, giving a range of scattering vector moduli (q = 4πinθ/λ) from 0.3 to about 3.5 Å^−1^, which correspond to a 1.8–25 Å d-spacing range. Furthermore, collagen structure was evaluated at the atomic level by recording two distinct signals: (i) The meridional reflection, the distance between adjacent amino acid residues along the central axis of the triple helix of collagen (periodicity index of the triple helix); (ii) the equatorial reflection, the distance between the triple collagen helices within the fibril (index of lateral packing). The analyses were carried out in duplicates, on collagen and collagen/PCL samples prepared as non-electrospun films, obtained by evaporation of 2 mL solution overnight and on the same electrospun samples.

##### Wettability Measurements

Wettability tests on nanofibrous membranes were carried out with a Cam tool 200 Contact Angle and Surface Tension Meter, KSV Instrument. The instrument was equipped with a telecentric camera and 55 cm focal length, monochromatic LED source, which allowed the measurement of the contact angle of a drop (volume 5 µL) left lay on the scaffold and dispensed by the needle of a syringe pump. Images were captured every second for 30 s. Before proceeding with the measurement, PCL and collagen/PCL nanofiber square pieces (2 cm^2^) were hydrated in water for 30 s and properly adhered on a coverslip. For each sample three replicates were done.

##### Mechanical Properties Evaluation

The mechanical properties of the nanofibers were measured with a TA.XT plus Texture Analyzer (Stable MicroSystems, Godalming, UK), equipped with a 5 kg load cell. Before testing, fiber thickness was measured by a Sicutool 3955 G-50 (Sicutool, Milan, Italy) apparatus. The nanofibers were cut (1 × 3 cm) and inserted between two clamps (A/TG grips probe) to expose a surface area of 1 cm^2^. The upper grip was raised at a trigger force of 1 mN under a controlled speed of 1 mm/s either to 30 mm or until the break point. Mechanical properties were evaluated in both dry and hydrated states (after 450 µL distilled water addition for 2 min). For each sample, the stress versus % elongation profile was recorded. From these curves the maximum elongation % was registered, while the maximum force was divided for the breaking area (Fmax). The Young modulus was calculated as slope of the initial linear portion of the curve. Five replicates were performed.

#### 2.2.7. Collagen Release Studies

Fiber samples of about 5 mg were weighed and inserted into a sterile plastic Petri dish with 7 mL of sterile PBS (phosphate buffer solution, pH 7.4) added with 1% penicillin–streptomycin–amphotericin solution (pen/strep/amph; Euroclone, Milan, Italy). Every 2 days, medium was replaced with fresh PBS. For each type of fiber three replicates were evaluated for weight loss at 7 days and for other three samples at 15 days. After 7 days, fibers were further evaluated to assess residual collagen amount by means of eosin staining and hydroxyproline determination. FTIR analysis was also performed. 

##### Eosin Staining

A 0.5% (w/v) eosin Y solution was used in 90% (v/v) ethanol. The test was performed by placing a portion of fiber on a slide and then adding a few drops of the eosin dye. Eosin was left to act for 2 s and then washed 4 times with an 80% (v/v) alcohol solution, and then twice with double-distilled water. The fiber was wetted with glycerol, covered with a cover glass and examined under an optical microscope and under CLSM microscope to evidence the eosin fluorescence (λ_ex_ = 524 nm, λ_em_ = 544 nm).

##### Hydroxyproline Quantification

Fiber samples of 1 mg were dissolved in 1 mL of acetic acid 90% (v/v) aqueous solution. From this solution 100 µL were transferred in 300 µL vials and the solvent was evaporated under nitrogen flow. The samples were then subjected to acid hydrolysis in 6M HCl, at 110 °C for 24 h in a nitrogen atmosphere, then examined by amino acid analysis, after precolumn derivatization with orthophthalate-aldehyde (OPA) and fluorenylmethylchloroformate (FMOC). The analysis of amino acids was carried out with a modular Jasco X-LC apparatus with fluorescence detector (λ_ex_ 340 nm, λ_em_ 446 nm for derivatives in OPA, λ_ex_ = 268 nm, λ_em_ = 308 nm for those in FMOC).

#### 2.2.8. Cell Adhesion and Morphology Study

Fibroblast morphological shape and adhesion to nanofibers were assessed by means of scanning electron microscopy (SEM) and confocal laser scanning microscopy (CLSM, Leica TCS SP2, Leica Microsystems, Milan, Italy). Briefly, nanofibers were cut in round pieces to get a surface area of 1.9 cm^2^ and UV irradiated for 15 min. The pieces were laid down into a 24-well plate and conditioned with 50 μL of complete medium (CM). One hundred thousand normal human dermal fibroblasts (NHDF) in 450 μL of CM were seeded in each well onto the nanofibers. After 3 and 7 days of culture, media was withdrawn, samples were washed with PBS and cells were fixed for 1 h at room temperature with 3% v/v of a glutaraldehyde solution. Subsequently, the samples were rinsed twice with 200 μL of PBS. For SEM analysis, samples were dehydrated with increasing concentration of ethanol (50%, 75%, and 100% v/v) and air dried at room temperature overnight. For CLSM analysis, samples were at first treated with Tryton 0.1% v/v for 5 min to permeabilize cell membranes to enable cytoskeleton staining. This was performed with 100 µL of Phalloidin-TRITC (concentration 50 μg/mL, Sigma, I) for 40 min, at room temperature and protected from light. Afterwards, substrates were washed twice with PBS and treated for 10 min, at room temperature and protected from light with 100 µL of Hoechst 33258 (Sigma, I) diluted in PBS 1: 10000, to stain the nuclei. Stained samples were then examined by CLSM and the fluorescence of Phalloidin TRITC and Hoechst 33258 (λ_ex_ = 346 nm, λ_em_ = 460 nm) were metered. The acquired images were processed using a “LAS X Life Science” software (Leika Microsystem, Milan, Italy).

#### 2.2.9. Biocompatibility Evaluation

NHDFs cells (Promocell GmbH, Heidelberg, G) from 5th to 10th passage were used. Cells were sub-cultured in complete medium (CM) consisting of Dulbecco’s modified Eagles Medium (DMEM) supplemented with 1% (v/v) antibiotic/antimycotic solution and 10% v/v inactivated fetal calf bovine serum (Sigma Aldrich, Milan, Italy). Nanofibers were cut in round pieces with a punch (Ø = 4 mm) and UV irradiated for 15 min. The pieces were placed into a 96-well plate and conditioned with 10 μL of CM. NHDFs (35.000 × well in 200 µL of CM) were seeded onto the nanofibrous membrane for 7 days. 

The cytotoxic effect of nanofibrous membranes was assessed by an MTT (3-(4,5-Dimethylthiazol-2-Yl)-2,5-Diphenyltetrazolium Bromide) test. First the medium was withdrawn and 50 µL per well of 7.5 µM MTT reagent in DMEM without red phenol was added in each well; subsequently the plates were placed in incubator at 37 °C for 3 h. Finally, the MTT reagent was removed and 100 μL of DMSO were added to each well to lyse the cellular membranes and to allow the complete dissolution of formazan crystals, derived from mitochondrial dehydrogenases reduction of the MTT dye. The absorbance was measured by means of an iMark® Microplate reader (Bio-Rad Laboratories Inc., Hercules, CA, USA) at a wavelength of 570 and 690 nm (reference wavelength) after 60 s of mild shaking. Eight replicates were performed for each sample. Results were expressed as % absorbance measured after contact with each sample with respect to that measured for CM.

## 3. Results and Discussion

### 3.1. Preparation of the Electrospun Nanofibers

#### 3.1.1. Rheological Studies

**Figure 1:**
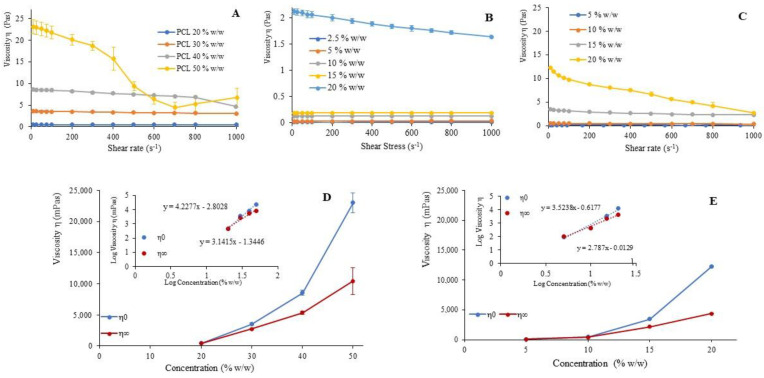
Rheological characterization of the polymer solutions. Viscosity (Pa.s) vs. shear rate (s^−1^) profiles of polymeric solutions prepared at different concentrations (% w/w) in 90% v /v acetic acid for poly-ε-caprolactone (PCL) (**A**), collagen (**B**) and collagen/PCL 1:1 (**C**) (mean values ± s.d.; n = 3). Average values of η0 and η∞ against concentration (a) and bi-logarithmic transformation of the same profiles (in the insert, b) evaluated for the solutions in 90% v/v acetic acid for PCL (**D**) and collagen/PCL 1:1 (**E**) (mean values ± s.d.; n = 3).

Figure 1A–C shows viscosity versus shear rate profiles measured for PCL, collagen and the 1:1 mixture solution. Collagen solutions show Newtonian behavior at all the tested concentrations but 20% (w/w). For PCL and for 1:1 mixture solution, a pseudoplastic behavior is more clearly evident at the highest concentrations. In the case of collagen/PCL 1:1 mixture, the viscosity curve at 20% (w/w) shows values quite higher than the curves of both PCL and collagen alone at the same concentration. This could be explained with hypothesis of the occurrence of hydrogen bonds between the carboxyl groups of PCL and the amide groups of collagen, as reported in literature by FTIR analysis [26].

On the basis of the viscosity values, considerations about the relationship between chain entanglement and polymer concentration can be made. The critical entanglement concentration (CEC) represents the lowest concentration at which the entanglement of the polymeric chains occurs; it depends on polymer molecular weight, chain conformation, and affinity to the solvent used [33]. As reported by Porter [34], at the CEC the passage from Newtonian to pseudoplastic behavior occurs. At this concentration, the parameter η_0_ that describes the polymeric rheological behavior when the sample is in unstressed condition, and η_∞_ representing the viscosity value of the polymer solution when the polymer chains are untightened by the high shear rates, are equal. For the different PCL and collagen/PCL concentrations, zero-shear viscosity (η_0_) and infinite shear viscosity (η_∞_) values are illustrated in Figure 1D,E. To better appreciate the concentration at which they became coincident, and the change between Newtonian to pseudoplastic behavior occurs, the log–log profiles of the viscosity parameters (η_0_ and η_∞_) vs. PCL or collagen/PCL concentration are given in the inserts. The same study is not reported for collagen solutions, because only at the highest concentration the pseudoplastic behavior could be seen. For PCL solutions, the estimated entanglement concentration in acetic acid 90% (v/v) aqueous solution corresponds to 22 % (w/w). When polymer is mixed with the protein, the passage from pseudoplastic to Newtonian behavior occurs at a lower concentration, of 7% (w/w). This change in behavior after the addition of the protein in collagen/PCL mixtures, can represent a further evidence of a possible interaction between the two components, in accordance with the previously observed increase of viscosity.

**Figure 2:**
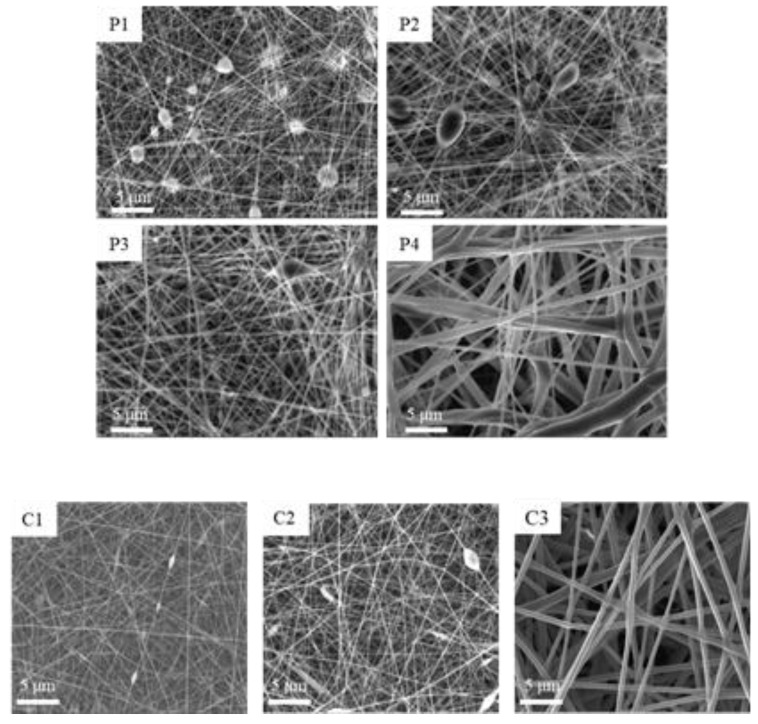
SEM photomicrographs of the electrospun nanofibers prepared from different concentrations of PCL (**P1**–**P4**) and Collagen (**C1**–**C3**), according to Table 1.

Different PCL formulations, in the range values of entanglement concentration previously obtained, were prepared in acetic acid 90% (v/v) aqueous solution and electrospun using the conditions indicated in Table 2. The SEM photomicrographs of the corresponding nanofibers are illustrated in Figure 2. Beads constitute the more common imperfections of electrospun membranes. The results obtained in the rheological studies are in this case confirmed. In fact, as evident in SEM photomicrographs of PCL formulations P1 and P2, below the entanglement concentration the electrospinning process led to non-continuous jet and collected fibers resulted rich in beads and with structural imperfections. These defects were not evident in nanofibers obtained by electrospinning of solutions above the entanglement concentration and continuous nanofibers were obtained from 20% (w/w) PCL (P3). Because 20% (w/w) concentration led to regular fibers also for collagen (C3) and was higher than the value calculated for collagen/PCL mixture, this final concentration was maintained for all the studied systems.

#### 3.1.2. Physical Properties of Collagen, PCL and Collagen/PCL Solutions

**Figure 3:**
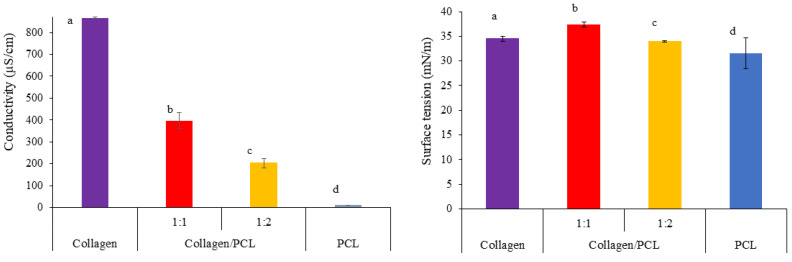
Conductivity (µS/cm) (on the left) and surface tension (mN/m) (on the right) values of collagen, collagen/PCL 1:1 and 1:2 (w/w) and PCL solutions prepared at total 20% (w/w) concentration. ANOVA 1 way—MRT (*p* < 0.05) (mean values ± s.d.; n = 5) for conductivity: a vs. b, a vs. c, a vs. d, b vs. c, b vs. d, c vs. d. ANOVA 1 way—MRT (*p* < 0.05) (mean values ± s.d.; n = 5) for surface tension: Not significant.

In Figure 3 the results of the physical characterization performed on the electrospun solutions are illustrated. As known, surface tension and conductivity solution parameters modulate the efficiency of the electrospinning technique by influencing the nanofiber’s homogeneity. Even if the solvent was the same in all cases, the conductivity values (µS/cm) highlighted the presence of strong statistically significant differences between collagen and PCL solutions. This was conceivable, as the protonated aminoacidic moieties of collagen, in fact, impacted significantly maintaining a high conductance while the non-ionic PCL did not contribute positively to conductivity. Intermediate conductivity values were recorded for the mixtures. Surface tension, on the contrary, was not influenced by the material employed. All the values were higher than the acetic acid 90% (v/v) aqueous solution (about 27 mN/m at 25 °C), but no statistical differences from collagen and PCL or the blends occurred.

#### 3.1.3. Design of Experiment (DOE) Based Evaluation of the Process Parameters

**Figure 4:**
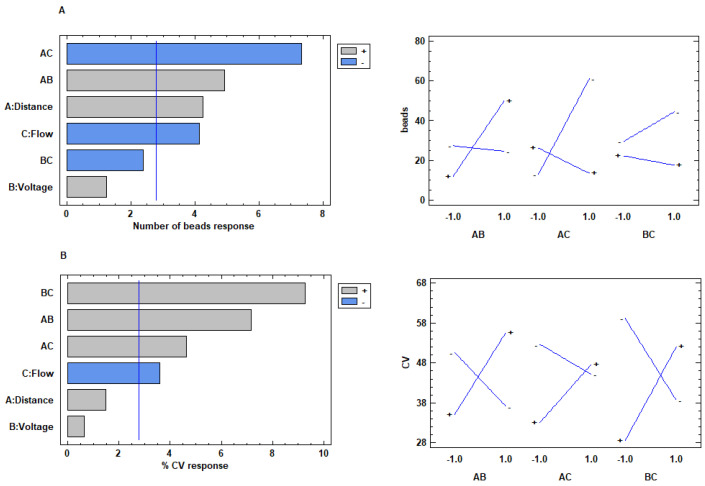
Effects of the considered process parameters (on the left) and interaction plots (on the right) for (**A**) “Number of beads” response for 1:1 (w/w) collagen/PCL fibers and (**B**) “Coefficient of variation (% CV)” response for collagen/PCL 1:2 (w/w) fibers.

The process parameters (flow, voltage, and distance) play an important role in the electrospinning technique, influencing the structure and morphology of the resulting nanofibers [38,39]. The influence of these parameters was investigated for both protein/polymer mixtures at 1:2 and 1:1 weight ratio on a statistical basis by full factorial experimental design (DOE). Electrospinning flow (ml/h), distance needle-collector (cm), and voltage (kV) were evaluated as factors. Nanofibers dimensions, number of beads into a fixed, standardized area and the coefficient of variation (% CV) of dimensions were set as response variables. This last parameter was chosen to describe the dimensional uniformity of the resulting fibers. The results obtained from the experimental design for the formulation collagen/PCL 1:1 showed a poor correlation, not statistically significant between the factors and both the responses dimensions and % CV. None process parameters, in the range considered, showed therefore a significant influence in obtaining small nanofibers and with a dimensionally homogeneous structure. Considering the presence of beads (Figure 4A), the distance needle-collector showed a positive and statistically significant effect. An increase in the distance was associated to an excessive stretching of the polymer solution jet. Due to the sudden solvent evaporation, the jet thus easily became discontinuous and instead of the electrospinning, an electrospray process occurred. On the other side, flow factor showed a negative and statistically significant effect on the development of irregular fibers rich in beads. An increase in flow led to a greater amount of solution on the tip of the needle decreasing the risk of a discontinuous and interrupted jet. The voltage did not show any statistically significant effect on the number of beads, however the interaction between distance and flow (AC) influences in negative and statistically significant way the response.

Therefore, to obtain continuous and regular fibers, it appeared useful to set both distance and flow at high level. Furthermore, the interaction between distance and voltage (AB) affected positively and significantly the number of beads, demonstrating how the increase of voltage, associated with increased distance, can compromise the efficiency of the process. 

For the formulations based on collagen/PCL 1:2 (w/w), both dimensions and number of beads responses showed a poor correlation with all the factors, that resulted not statistically significant. When coefficient of variation (% CV) was considered as response (Figure 4B), a negative and statistically significant effect of the flow on the dimensional homogeneity of the nanofibers was evident, while the distance needle-collector and the voltage applied did not cause a statistically significant effect. Given this evidence, the flow factor should be set up at the higher level to obtain fibers dimensionally homogeneous. All the binary interactions showed significant positive effect. As previously observed, when voltage and distance are set up at high levels, a jet instability occurs, no continuous fibers can be collected, and the electrospinning process becomes irregular. The interaction between distance and flow (AC) demonstrates that the increase in both flow and distance can reduce the uniformity of the fibers. 

### 3.2. Characterization of Nanofibrous Membranes Based On Collagen/PCL Mixtures 

**Figure 5:**
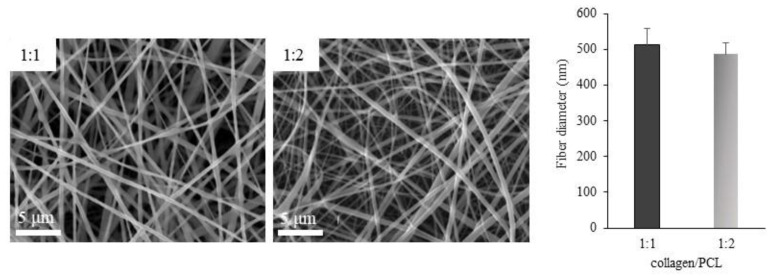
Morphological (on the left) and dimensional (on the right) characterization of the nanofibers based on collagen/PCL mixtures 1:1 and 1:2 (w/w).

The solutions based on collagen, PCL and collagen/PCL at two different weight ratios (1:1 and 1:2) were therefore electrospun by setting the process parameters according to the results of the DOE based development phase. Electrospinning parameters were set at 25 cm distance, 20 kV voltage and 0.8 mL/h flow for collagen/PCL 1:1 (w/w), and at 20 cm distance, 20 kV voltage and 0.8 mL/h flow for collagen/PCL 1:2 (w/w). Homogeneous and reproducible nanofibrous membranes were collected, as illustrated in Figure 5, and further characterized. Although the most restrictive definition of nanoscale considers 100 nm as the upper limit, both the obtained samples show dimensions comparable with those of structures that in the literature are commonly classified as nanofibers.

#### 3.2.1. FTIR Analysis

**Figure 6:**
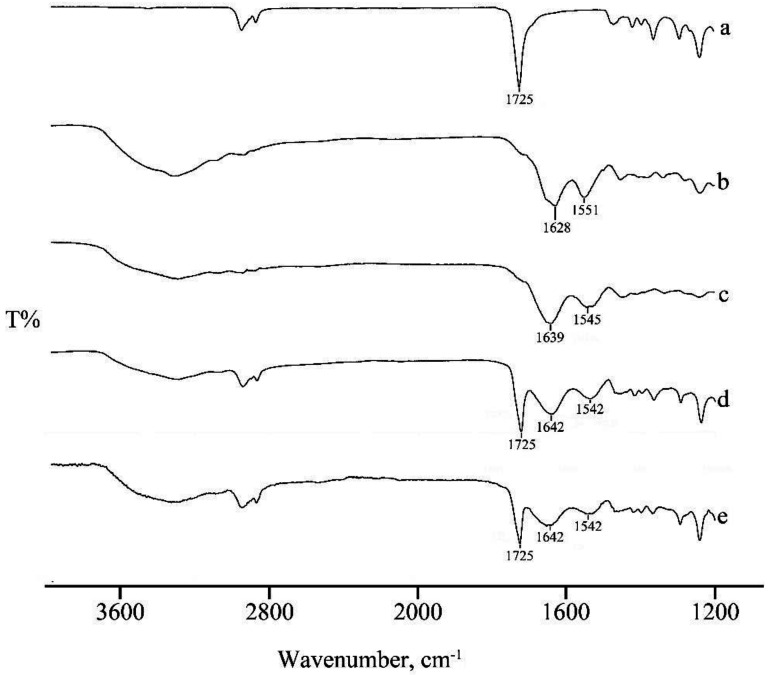
FTIR spectra of PCL (**a**), native collagen freeze dried (**b**) and nanofibers (NFs) based on collagen (**c**), collagen/PCL 1:1 (**d**) and 1:2 (**e**) (w/w).

In Figure 6, FT-IR spectra of PCL and native collagen are compared with spectra of electrospun collagen and collagen/PCL electrospun nanofibers at both the 1:1 and 1:2 weight ratios. A clear shift in the characteristic bands of the amidic bonds from native collagen (1630 and 1550 cm^−1^) and collagen nanofibers (1640 and 1545 cm^−1^) was detectable, in accordance with the literature [24] and indicating that, with respect to the raw material, protein conformation was affected during electrospinning process, conceivably both by dissolution in strongly acidic medium and by the high shears used during electrospinning. Another shift in amide signal was recorded when PCL was added to the electrospun formulation (1640 and 1543 cm^−1^), in both weight ratios suggesting an effective interaction between the two components, as commonly reported in literature [21,26], and in accordance with the results of the rheological characterization.

#### 3.2.2. WAXS Analysis

**Figure 7:**
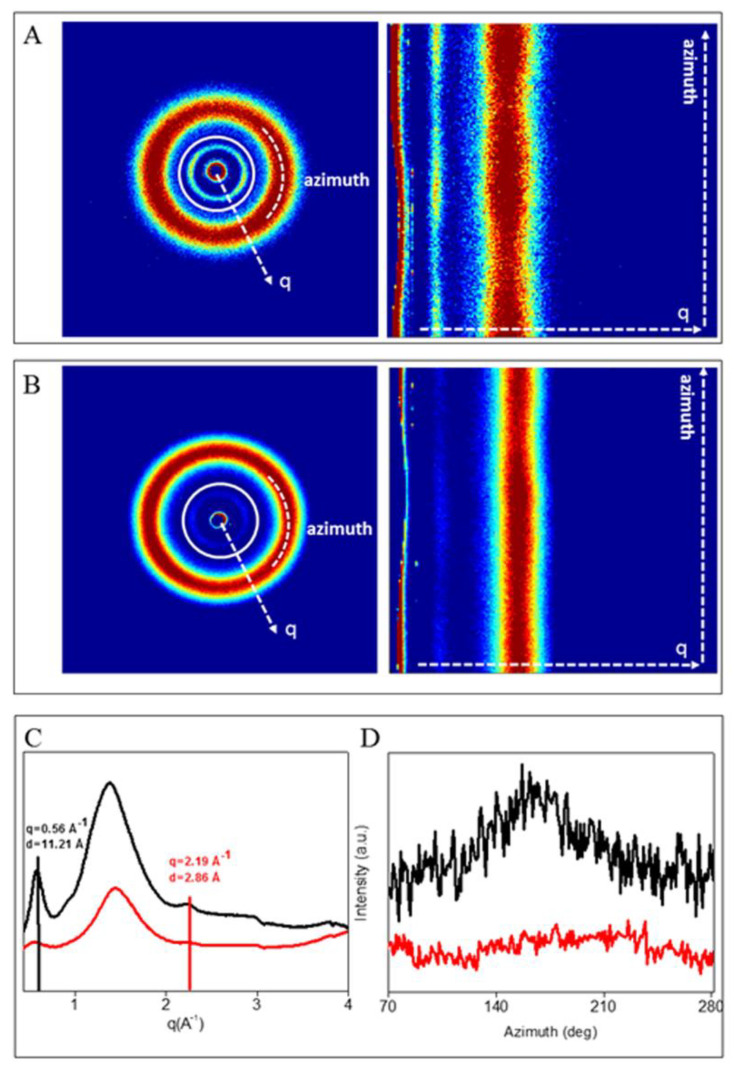
Diffracted intensity distribution of collagen in native form (**A**) and treatment with acetic acid 90% (**B**). (**C**) 1D Wide Angle X-ray Scattering (WAXS) profiles of collagen in native form (black profile) and collagen treated with acetic acid 90% v/v (red profile). (**D**) Azimuthal profiles along equatorial direction extracted at q= 0.56 Å^−1^ for both samples: native collagen (black profile) and acidic collagen (red profile).

**Figure 8:**
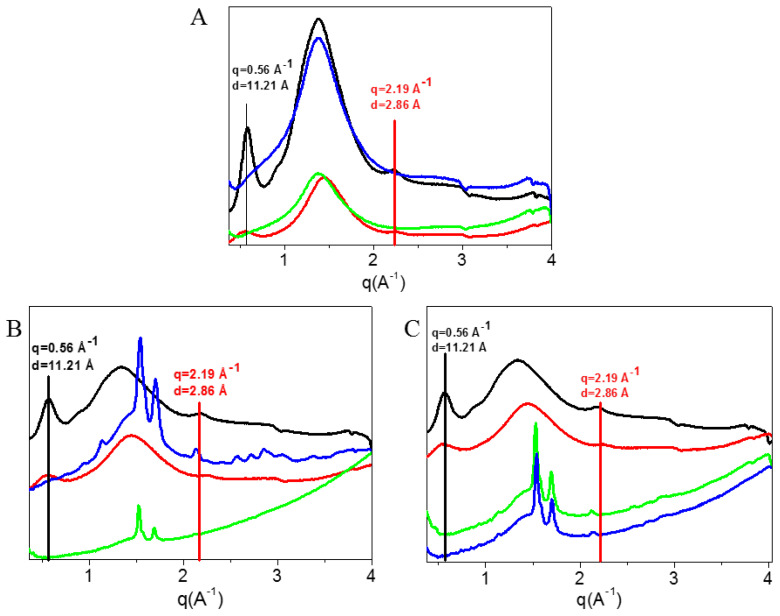
WAXS 1D profiles obtained from radial integration of 2D diffraction patterns. (**A**) WAXS spectra of native collagen (black), collagen in 90% (v/v) acetic acid aqueous solution (red) and collagen electrospun nanofibers with different weights 15 mg (green) and 55 mg (blue) (protein concentration in all samples: 20% w/w). (**B**) WAXS spectra of native collagen (black profile), collagen in 90% (v/v) acetic acid aqueous solution (red profile) and collagen/PCL mixtures with 1:2 weight ratio (green profile) and 1:1 weight ratio (blue profile) weight ratio respectively, before the electrospinning. (**C**) WAXS spectra of native collagen (black profile), collagen in 90% (v/v) acetic acid aqueous solution (red profile) and collagen/PCL electrospun nanofibers with 1:2 weight ratio (green profile) and 1:1 weight ratio (blue profile), after the electrospinning treatment.

Degradation of collagen during electrospinning process is commonly described in the literature, although different findings are reported depending on the electrospinning solvent used. In perfluorated solvents (e.g., HFP) an extensive denaturation of collagen during the electrospinning process was evidenced by some authors [14], while others observed some triple helical structure preservation as resulted by circular dichroism (CD) spectra [40]. Based on CD analysis, Liu et al [41] found lower loss of triple helical structure using 40% (v/v) acetic acid aqueous solution as electrospinning solvent than using HFP. To better investigate this aspect for the here selected electrospinning conditions, WAXS spectra were recorded for native freeze-dried collagen, before and after acidic dissolution in acetic acid 90% v/v (Figure 7), after electrospinning treatment (Figure 8A), and in blend with PCL in collagen/PCL nanofibers (Figure 8B,C). Indeed, in recent works [42,43,44] the successful applicability of this technique on multiscale structural characterization of collagen, starting from raw macromolecules to the final biomaterials, was demonstrated.

WAXS analysis permits to evaluate the atomic scale configuration and arrangement of collagen molecules, providing information about the triple helical structure along the two main directions: meridian (molecular axis) and equator, orthogonally oriented with respect to the meridian. As widely described in literature [45,46], two collagen’s characteristic diffraction signals are generally recognized in the diffraction pattern along the above mentioned directions: the meridional signal, describing the distance between two adjacent amino acids along the c-axis of the molecule; and the equatorial signal, related to the lateral packing of the triple helices within the supramolecular structure. An additional third amorphous-like signal is commonly detected between them, linked to the distance between triple helical skeletons in the supramolecular arrangement [47].

The q values of the three diffraction peaks and the correspondent d positions in the direct space, thus the molecular spacings, for native collagen 1% v/v from bovine origin are shown in Table 3.

As shown in Figure 7A (native collagen) and 7B (collagen dissolved in 90% (v/v) acetic acid aqueous solution), the equatorial reflections, marked in the white circle, were recognized in both samples. However only in the native collagen (7A) the presence of a non-continuous ring, by two oriented arcs located along the equator, are detected, suggesting a slightly signal orientation that in the direct space corresponds to a reduced but still maintained lateral packing organization of triple helices. On the contrary, in the acidic collagen, the presence of the equatorial full ring signal is a marker of molecules random organization in the sample.

In order to quantitative evaluate both the structural integrity of the helical structure (meridional signal) and lateral packing of collagen molecules (equatorial signal) before and after the acidic dissolution, the 2D WAXS patterns were radially integrated, obtaining the 1D profiles showed in Figure 7C. The two collagen’s characteristic peaks, the meridional at q = 2.19 Å^−1^, corresponding to an axial periodicity of d = 2.8 Å in the direct space, and the equatorial at q = 0.56 Å^−1^, corresponding to a lateral packing distance between molecules of d = 11 Å, are recognized. Although the diffracted intensity of the profiles are not comparable as they are dependent to the thickness and density of the samples that in our case were powders, thus with high variability of both thickness and density parameters, the relative evaluation and comparison can still be performed. In particular, after the acidic treatment the meridional reflection (marker of the triple helical integrity) is barely visible, suggesting a lower triple-helix conformation and organization, due to the decreased axial crystallinity of collagen solved in 90% (v/v) acetic acid aqueous solution compared to the native protein. The degree of fiber order was also investigated integrating the 2D WAXS patterns along the azimuth (Figure 7A,B, white arcs) in correspondence of the equatorial peak (q = 0.56 Å^−1^). 

The azimuthal profiles (Figure 7D) were fitted by a Gaussian function to evaluate the Full-Width-at Half-Maximum (FWHM). The results (Table 4) showed a higher extent of crystalline domain (smaller FWHM) in the native collagen (Figure 7D, black profile), than in the acidic one (Figure 7D, red profile). 

It is worth noting that, despite the triple helices are not clearly oriented within the native collagen, they still retain the “memory” of the natural molecular organization, thus the lateral packing distance and the axial periodicity of triple helices. This could be probably due to retention of the lateral crosslinks and H-bonding between NH group of glycine and the backbone CO group of a residue in the X position of the neighboring chain. Conversely, the loss of spatial order in collagen treated with acetic acid 90% v/v, highlights that the acidic dissolution induces the disruption of triple helical lateral packing, promoting randomly oriented fibers in the sample. Thus, the findings suggest that the acidic treatment of the protein negatively affects not only the helix along its molecular axis, but also the lateral packing and the supramolecular assembly of collagen molecules.

Furthermore, the evaluation of the effect of the electrospinning process on the protein structure stability was performed by comparing the diffraction profiles of collagen in 90% (v/v) acetic acid aqueous solution before and after the electrospinning process (Figure 8A). Collagen based electrospun membranes with different weights were characterized (Figure 8A (green profile) 15 mg, 8A (blue profile) 55 mg). In both profiles, the meridional and the equatorial reflections almost disappeared after electrospinning process, thus, the triple helix structure is no longer visible. Moreover, a further decrease in molecular lateral packing, stronger that already observed after acidic treatment, occurs. Figure 8B,C show the effect of blending with PCL, in solution and after electrospinning, respectively. The profiles of native collagen and of collagen in acetic acid are repeated for comparison purposes. Figure 8B shows that the integrity of the triple helix structure of collagen is already lost when collagen is blended with polycaprolactone in 90% (v/v) acetic acid aqueous solution at both different weight ratios (Figure 8B (green profile) 1:2 w/w, 8B (blue profile) 1:1 w/w). In both mixtures, the meridional and equatorial reflections are not identified, indicating the substantial denaturation of the protein. Figure 8C confirms that after electrospinning process, in both membranes (Figure 8C (green profile) 1:2 w/w, 8C (blue profile) 1:1 w/w) the meridional and the equatorial reflections were absent. The additional peaks shown in the nanofibrous membranes, near the meridional reflection region, were typical of the PCL.

#### 3.2.3. Wettability

**Figure 9:**
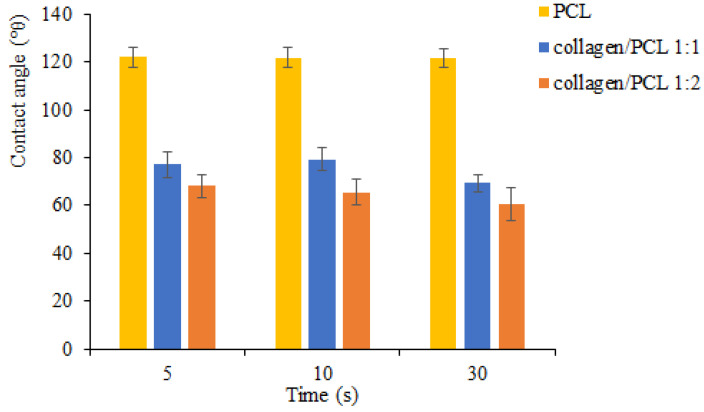
Comparison of contact angle values for PCL and collagen/PCL nanofibers at two weight ratios (1:1 and 1:2) (mean values ± s.d. n = 3).

Figure 9 shows the results of wettability analyses for PCL and collagen/PCL (1:1 and 1:2 w/w) electrospun nanofibers. As expected, in accordance with the literature, the addition of collagen in the nanofibers involved a substantial reduction of the contact angle values compared to the nanofibrous membranes obtained with PCL only. The wettability increased thanks to the presence of the hydrophilic protein which reduced the interfacial tension between the water and the solid surface of the nanofibers. Quite close results characterize the two samples with lower and higher amount of collagen.

#### 3.2.4. Mechanical Properties

Table 5 reports the mechanical properties results of the membranes as tensile strength (MPa), strain at the break point and Young’s modulus (MPa) values, measured in dry and hydrated state. Young’s modulus was calculated as the slope of the linear section of the stress–strain curve where the proportional relationship between stress and strain is described by Hooke’s law. All the results obtained are in line with what reported in literature [48,49]. The tensile strength was higher for all the samples when evaluated in the dry state. Collagen membranes present stress-strain curves typical of brittle materials linear over their full range of strain, eventually terminating in fracture without appreciable plastic flow and with a significantly higher strength associated to a lower strain, when compared to the other samples enriched with PCL. The addition of PCL at 1:1 weight ratio to the formulation drastically decreased tensile strength together with the Young’s modulus and a more fragile material, with poor deformability, was obtained. The further decrease of collagen in the sample collagen/PCL 1:2 (w/w) led to more flexible mats showing a considerable increase in strain together with a slight increase in tensile strength. Fibers containing PCL, compared to other nanofibrous membranes, were characterized by high plasticity with lower strength and higher deformation. The mechanical behavior in the hydrated state was evaluated to simulate use condition in a biological environment. The results of collagen fibers are not reported as they undergo a complete dissolution in the aqueous medium. In all the hydrated membranes, a decrease in tensile and a significant increase in elongation, therefore in strain values, occurred. For collagen/PCL 1:1 (w/w) membranes, the combination between the highest content of collagen with the more plastic PCL led to nanofibers with the highest capability of deformation. Lower Young’s modulus values were registered at the hydrated state. A deviation from the linear proportionality occurred, that is usually associated with a stress-induced plastic flow. The microstructural rearrangements, associated to a plastic behavior, lead to a typical non-reversible event when the load is removed, and the elastic limit is often completely reduced. The nanofibers mechanical properties can be affected by lot of factors such as dimensions, thickness of the membrane and fiber deposition method [50]. Some reports considered that thinner fibers have higher elasticity modulus than thicker ones [51]. In the present case, however, as fibers have quite similar dimensions and the same random deposition, the differences in mechanical strength seem to be related mostly to the composition.

#### 3.2.5. Morphological Stability of Nanofiber Structure in the Hydrated State

**Figure 10:**
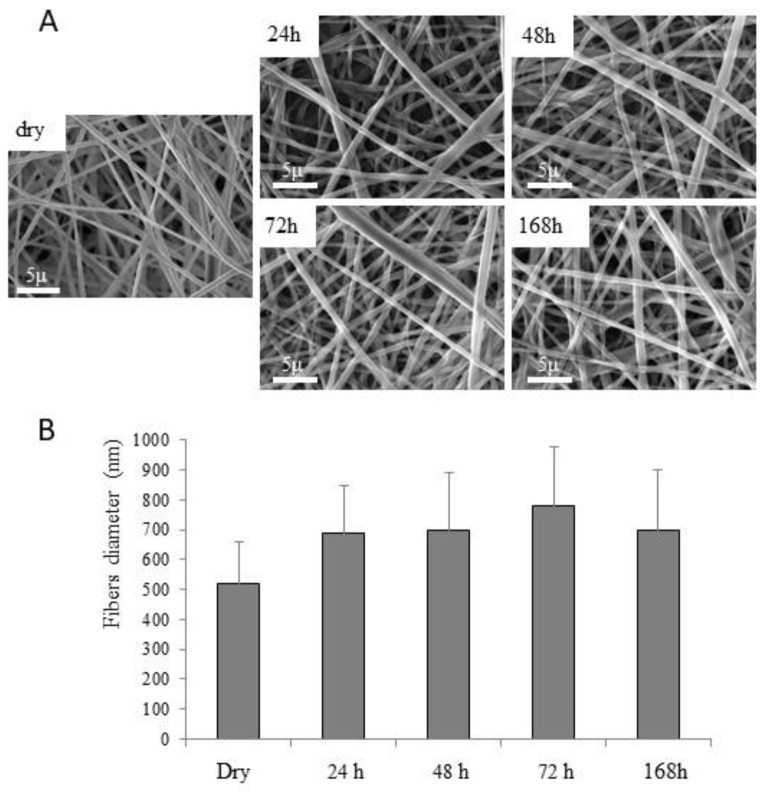
Photomicrographs SEM (**A**) of 1:1 weight ratio collagen/PCL nanofibers in dry and hydrated states from 24 h up to 1 week (168 h) at room temperature, and size diameters comparison (**B**) (mean values ± s.d., n = 60).

The morphological stability in the hydrated state evaluated for the collagen/PCL nanofibers is reported in Figure 10 for the collagen/PCL 1:1 (w/w) formulation. From 24 h up to 1 week of immersion in deionized water, the architectural structure of nanofibers was preserved, and only a slight and not significant increase of fiber diameters occurred. Similar results were recorded for collagen/PCL nanofibers 1:2 weight ratio (data not shown).

### 3.3. Collagen Release

It is well known from the literature that the biodegradation of PCL takes quite long time, until about 90 days [27]. This is in accordance with the morphological stability observed for both the mixture nanofibers characterized in the present work. Less clear is from the literature the rate of leaching for the collagen component of nanofibers based, such in the present case, on collagen and PCL mixtures.

**Figure 11:**
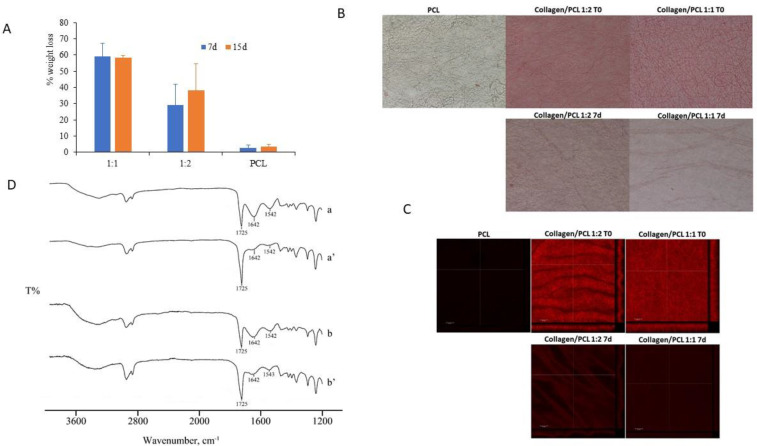
Collagen release from the nanofibrous membranes. (**A**) Weight loss (%) after 7 and 15 days in PBS. (**B**) collagen staining with eosin, optical microscope images, at time 0 and after 7 days in PBS. (**C**) collagen staining with eosin, CLSM images, at time 0 and after 7 days in PBS. (**D**) FTIR spectra at time 0 (a: collagen/PCL 1:1, b: collagen/PCL 1:2) and after 7 days in PBS (a’: collagen/PCL 1:1, b’: collagen/PCL 1:2).

Some authors found a quite low weight loss, corresponding to about 7.6%, for PCL:collagen at 2:1 weight ratio after a 24 h in water/EtOH [21]. This result is explained with the interactions, based on hydrogen bonds, observed between PCL and the protein. The results here obtained seem in line with the quite a fast leaching of most of the collagen present in PCL/collagen 9:1 nanofibers observed by Dulnik et al. [27]. In the present work the results obtained after one week of hydration in PBS at 37 °C are illustrated in Figure 11. The weight loss of the fibers is illustrated in Figure 11A, confirming the low degradation rate of PCL and suggesting an almost complete leaching of collagen. A qualitative confirmation of this result is illustrated in Figure 11B,C, where the collagen is stained with eosin and visualized at the optical and confocal microscopy.

The FTIR spectra of the same membranes after 7 days of hydration in PBS at 37 °C are reported in Figure 11D. The spectra of the fibers before the test are reported again to make the comparison easier. It is possible to appreciate a decrease of the bands at 1640 cm^−1^ and at 1542 cm^−1^ after the release test. A semi-quantitative analysis was performed by the software provided with the ATR-FTIR equipment, integrating the band at about 1640 cm^−1^ by comparison with the PCL band taken as reference, considering its very low degradation rate. This evaluation suggested that about 8% of collagen was still present in collagen/PCL 1:1 (w/w) sample, and about 25% in the collagen/PCL 1:2 (w/w) sample. All these results were confirmed by collagen quantification through hydrolysis and hydroxyproline determination in the fibers after 7 days in aqueous environment, that resulted in a 12% (± 0.9) of the initial amount of collagen still present for the collagen/PCL 1:1 (w/w) sample, and in a 22% (± 1.9) for the collagen/PCL 1:2 (w/w) sample. A slightly lower percentage in leaching can be observed for the collagen/PCL 1:2 (w/w) than for the 1:1 (w/w) sample, probably for the higher possibility of interaction/miscibility of the collagen when the highest PCL ratio is present [21,27].

### 3.4. Fibroblast Growth and Adhesion

**Figure 12:**
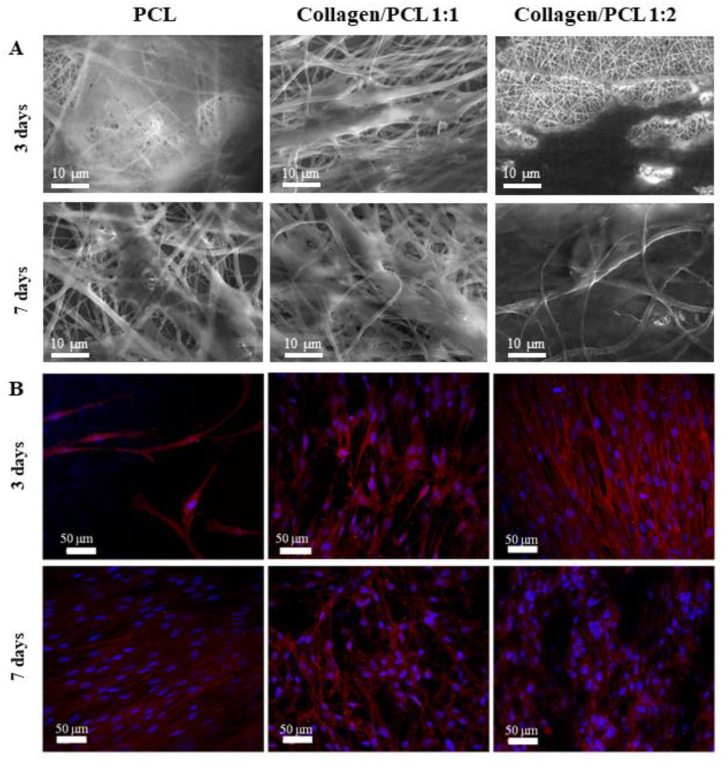
Fibroblasts growth and adhesion on nanofibers after 3 and 7 days culture. (**A**) SEM photomicrographs (**B**) confocal laser scanning microscopy (CLSM) images (nuclei blue marked with Hoechst, cytoskeleton red marked with Phalloidin-TRITC).

Figure 12 shows the growth and adhesion at 3 and 7 days of normal human dermal fibroblasts (NHDF) on the nanofibers based on PCL, collagen and on their mixtures (1:1 and 1:2 weight ratio). A beneficial effect of collagen addition on the growth of cells onto the fibrous membranes is related to the presence of Arg-Gly-Asp (RGD) sequence in polypeptide molecules, a tripeptide that plays an important role in mediating cell adhesion interactions. RGD sequence, found in many extracellular matrix proteins, is recognized by integrins and acts as cell binding site [7]. SEM photomicrographs (magnification 10 kx) of cellular substrates grown on nanofibrous membranes for 3 days and 7 days are shown in Figure 12A. An extended cell adhesion was evident for all the formulations. In all cases fibroblasts were spread all over the surface reaching the confluence. The typical cell morphology was preserved, and cells appeared deeply incorporated into the membrane, in particular, in the case of collagen/PCL nanofibers 1:1 weight ratio. CLSM photomicrographs of cellular substrates grown for 3 and 7 days (labelled nuclei in blue with Hoerchst 33582 and cytoskeletons marked in red with phalloidin TRITC) on PCL, collagen/PCL 1:1 and 1:2 weight ratios confirmed the quantitative data of viability obtained by the MTT test and the cell morphology presented in SEM photomicrographs (Figure 12B). In all cases, cells were numerous, adhered to the membranes and with a healthy morphology. 

### 3.5. Collagen vs. Gelatin Fibers Characterization

Previous studies reported in the literature demonstrated the denaturation of collagen in electrospun nanofibrous membranes, leading to the occurrence of gelatin-based scaffolds [14]. This occurrence was observed when electrospinning was performed in fluorinated solvents, or with acetic-formic acid mixtures [24,28]. To more directly understand the role of collagen, even after denaturation, with respect to gelatin, gelatin/PCL nanofibers were obtained from solutions prepared in 90% (v/v) acetic acid aqueous solution at the same weight ratios previously considered for collagen. The same process parameters employed for collagen/PCL nanofibers as suggested by DOE study were used. Mean dimensions between the two formulations were comparable, resulting 862.1 ± 181.7 nm for the 1:1 (w/w) sample, and 800.8 ± 336.9 nm for the 1:2 sample. 

**Figure 13:**
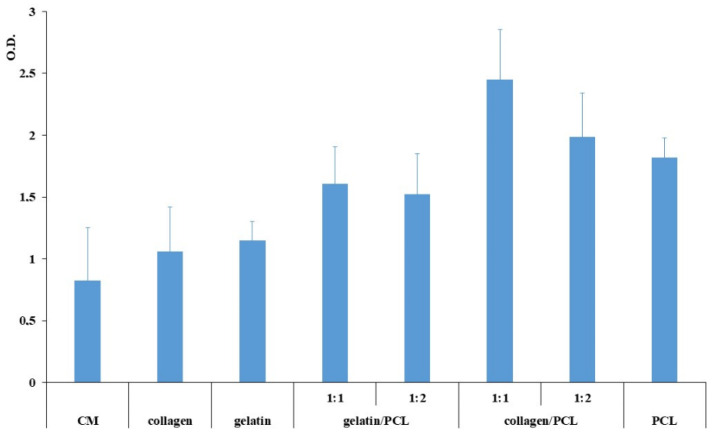
Cell viability (Optical density O.D.) after 7 days of normal human dermal fibroblasts (NHDF) seeded on nanofibrous membranes based on collagen, gelatin, gelatin/PCL, collagen/PCL and PCL (ANOVA 1 via—MRT (*p* < 0.05) mean values ± s.d., n = 8). Statistically significant differences: Gelatin/PCL 1:1 (w/w) and 1:2 (w/w) vs. control (CM), collagen/PCL 1:1 (w/w) and 1:2 (w/w) vs. control, PCL vs. control, collagen/PCL 1:1 (w/w) vs. gelatin/PCL 1:1 (w/w), collagen/PCL 1:1 (w/w) vs. PCL, Collagen/PCL 1:1 (w/w) and 1:2 (w/w) vs. collagen, gelatin/PCL 1:1 (w/w) and 1:2 (w/w) vs. gelatin.

The cell biocompatibility results are illustrated in Figure 13 for nanofibrous membranes based on collagen, gelatin, collagen/PCL, and gelatin/PCL at two different weight ratios (1:1 and 1:2) after 7 days. A significantly higher growth was evident for all nanofibrous membranes containing the insoluble polymer (PCL) compared to the control and to the soluble membranes based on collagen and gelatin. The positive effect of PCL membrane can be partially attributed to the mechanism of support of the tridimensional structure of the slowly degradable PCL, that in fact also alone results in a significant increase of cell viability. The comparison between the fibers based on collagen and on gelatin shows that, although only for 1:1 weight ratio the difference was found statistically significant, cell proliferation was higher for the collagen/PCL fibers than for the corresponding gelatin-based samples, demonstrating that in spite of the triple helix loss, collagen maintained better interaction capacity with the cells than gelatin, in accordance with literature results showing more efficient cell growth stimulation for collagen than for gelatin [28]. Only the fibers based on the higher collagen/PCL ratio showed a statistically significant improvement of viability in comparison with PCL fibers. Considering the small volumes of fluid at the wound bed, collagen, although released from the fibers, is conceivably made available for the cells within the fiber network during the period of the test, showing a clear concentration dependent positive effect. 

Tensile strength (MPa) and Young’s modulus (MPa) were evaluated on gelatin/PCL 1:1 membranes, to highlight any differences between collagen and gelatin on mechanical properties both in dry and hydrated states. In the dry nanofibrous membranes based on gelatin/PCL, tensile strength and Young’s modulus were 1.03 ± 0.09 MPa and 0.34 ± 0.001 MPa, slightly lower than those of collagen/PCL fibers, and a similar maximum elongation % (4.8± 0.7). The same was observed in the hydrated state, where gelatin/PCL fibers showed a tensile strength of 0.54 ± 0.03 MPa a Young’s modulus of 0.04 ± 0.001 MPa and a maximum elongation % of 175.7 ± 21.8. In collagen/PCL nanofibers the combination of the plastic deformation of the synthetic polymer and the protein high elastic module appears promising to follow the body movements and enhance wound healing even in case of application on joints and articulations. These data are in accordance with the evidence reported in literature, where scaffolds based on collagen/PCL seemed preferable for in vivo implantation, appearing smooth and glossy with good elasticity, while the gelatin/PCL electrospun scaffolds were relatively soft and easily collapsible [18].

## 4. Conclusions

Nanofibrous membranes based on collagen and PCL alone or combined were successfully obtained by electrospinning in 90% (v/v) acetic acid aqueous solution as solvent. The preliminary development phase indicated that all the tested solutions had very similar surface tension. Conductivity was on the other hand strongly variable, ranging from the very low values of the non-ionic PCL solution, to the high values observed with the increase of collagen concentration in the mixtures. No influence of conductivity on solution spinnability was however evidenced in the present study. The DOE approach indicated that, among electrospinning process parameters, the distance of the collector and the electrospinning flow rate play highest relevance on nanofiber homogeneity and regular dimensions. It was also confirmed that DOE represents a powerful methodology to put in evidence on a sound statistical basis the effect of critical process parameters on the desired characteristics of a drug delivery system. In the present case, moreover, it allowed to evidence a strong effect of interactions, that must be considered in setting on a rational base the apparatus conditions. Both mixtures at 1:2 and 1:1 collagen/PCL weight ratios showed improved mechanical properties in the hydrated state, with respect to both collagen and PCL alone. For both collagen/PCL formulations an excellent biocompatibility was highlighted, associated with an increase in proliferation and with a good cell adhesion. Significantly higher proliferation was obtained in the case of the highest content of collagen at one week, demonstrating that the protein positively influenced the cell growth stimulation of the scaffolds, although at the same time a complete release of the collagen component of the fibers was observed. WAXS analyses on collagen and collagen/PCL fibers confirmed the loss of the triple helix structure due both to the strongly acidic solvent and to the high shear of electrospinning process. However, the comparison between collagen/PCL and gelatin/PCL electrospun nanofibers in term of mechanical behavior and biocompatibility showed better results for collagen-based nanofibers, characterized by higher mechanical strength and enhancement in fibroblast proliferation, especially for the 1:1 weight ratio. Therefore, collagen/PCL nanofibrous membranes with the highest amount of protein seem to be promising supports for cell proliferation and for a possible application in wound healing.

## Figures and Tables

**Table 1:** Experimental points of the full factorial design.

Sample	Distance (cm)	Voltage (kV)	Flow (ml /h)
C.P.	0	0	0
1	−1	−1	−1
2	+1	−1	−1
3	−1	+1	−1
4	+1	+1	−1
C.P.	0	0	0
5	−1	−1	+1
6	+1	−1	+1
7	−1	+1	+1
8	+1	+1	+1
C.P.	0	0	0

**Table 2:** Mean fiber dimensions of PCL (P1-P4) and collagen (C1-C3) electrospun formulations.

Formulation	Concentration (% w/w)	Process Parameters	Fiber Dimensions (nm)
P1	10	20 Kv-15 cm-0.8 mL/h	110.93 ± 29.40
P2	15		197.08 ± 58.55
P3	20		211.60 ± 59.51
P4	30		494.80 ± 93.30
C1	10	25 kV-15 cm-0.4 mL/h	175.55 ± 59.21
C2	15	25 kV-15 cm-0.4 mL/h	159.63 ± 53.01
C3	20	25 kV-15 cm-0.4 mL/h	668.24 ± 152.42

**Table 3:** Equatorial, meridional, and diffuse reflections of the native soluble collagen.

Diffraction Signal	q (Å^−1^)	D(Å)
Equatorial Refl	0.56 ± 0.02	11± 0.03
Meridional Refl	2.19 ± 0.02	2.8± 0.03
Diffuse Scatter	0.8 < q < 2.09	7.8 < d < 3

**Table 4:** Full-Width-at Half-Maximum (FWHM) of 1D azimuthal intensity profiles of the equatorial diffraction peak (q = 0.56 Å^−1^) for both native and acidic collagen.

Sample	q (Å^−1^)	FWHM (Å)
Native collagen	0.56 ± 0.02	93.3± 3.6
Native collagen in acetic acid 90% v/v	0.56 ± 0.02	160.0± 11.04

**Table 5:** Tensile strength (MPa), maximum strain and Young’s modulus (MPa) values of nanofibrous membranes evaluated at the dry and hydrated states. (mean values ± s.d., n = 6).

DRY	Tensile Strength (MPa)	Elongation (%)	Young’s Modulus (MPa)	HYDRATED	Tensile Strength (MPa)	Elongation (%)	Young’s Modulus (MPa)
Collagen	4.06 ± 0.05	33.2 ± 13.1	1.26 ± 0.1	Collagen	/	/	/
Collagen/PCL 1:1 (w/w)	2.50 ± 0.03	12.8 ± 0.8	0.58 ± 0.06	Collagen/PCL 1:1 (w/w)	0.89 ± 0.01	356.2 ± 45.9	0.04 ± 0.01
Collagen/PCL 1:2 (w/w)	3.53 ± 0.13	63.3 ± 8.7	0.10 ± 0.01	Collagen/PCL 1:2 (w/w)	2.75 ± 0.18	169.0 ± 22.5	0.06 ± 0.01
PCL	2.15 ± 0.13	140.5 ± 55.5	0.05 ± 0.01	PCL	0.96 ± 0.07	230.4 ± 20.3	0.01 ± 0.001
